# Divergent effects of pitch feedback on online and offline motor sequence learning

**DOI:** 10.3389/fnbeh.2025.1680277

**Published:** 2025-10-24

**Authors:** Pauline Ploettner, Christoph Muehlberg, Felix Psurek, Christopher Fricke, Jost-Julian Rumpf

**Affiliations:** Department of Neurology, University of Leipzig Medical Center, Leipzig, Germany

**Keywords:** motor sequence learning, motor learning, motor memory consolidation, sensory feedback, auditory feedback, action-perception coupling

## Abstract

**Introduction:**

Motor sequence learning - the integration of individual movement elements into coordinated actions - is essential for everyday skills. This process comprises online learning during practice and post-practice offline consolidation. A key mechanism is action–perception coupling, in which motor actions become linked with predictable sensory outcomes. Pitch feedback, which conveys timing and spatial information, may strengthen this coupling and facilitate skill acquisition. Here, we evaluated pitch feedback as a tool to modulate both online and offline motor sequence learning.

**Methods:**

We included sixty healthy young non-musicians (mean age: 28.4 ± 4.6 years) who were asked to perform a finger-tapping task on a MIDI keyboard. They were randomly assigned to one of three auditory feedback groups: congruent, fixed, and random pitch feedback. The task involved repeatedly performing an 11-item sequence with the right hand. Pitch feedback was delivered according to group assignment during 14 training blocks of six sequences each. Prior to training, participants completed one block of the task without pitch feedback to assess baseline performance. Retention was tested 6 h later under two conditions: seven blocks without pitch feedback (Retest 1) and seven blocks with pitch feedback (Retest 2).

**Results:**

Congruent pitch feedback facilitated online learning across the initial training session compared to fixed or random feedback. This advantage of congruent pitch feedback persisted during retesting in the presence of feedback (Retest 2), but did not generalize to task performance in the absence of pitch feedback (Retest 1). Importantly, while online learning and task performance were facilitated by congruent pitch feedback, between-session performance changes were significantly larger in the group that received random pitch feedback during the initial training session compared to the congruent and fixed feedback groups.

**Conclusion:**

These findings highlight a dissociation between feedback types that optimize immediate performance and those that promote lasting motor memory formation. While congruent pitch feedback facilitates online skill acquisition compared to fixed or random pitch feedback, unpredictable auditory input may challenge learners to engage internal monitoring mechanisms, leading to more robust, feedback-independent motor memory consolidation. These insights have implications for optimizing auditory feedback in motor learning and neurorehabilitation contexts.

## Introduction

Motor sequence learning - the process of acquiring and integrating individual movements into smooth, coordinated sequential actions - is essential for everyday activities from typing or using a smartphone to playing a musical instrument. This form of skill acquisition involves both online learning during active practice and offline motor memory consolidation, during which training-induced performance gains are stabilized or even enhanced in the absence of further practice (Dayan and Cohen, 2011; Walker et al., 2003). During online motor sequence learning, movement elements of the sequence become increasingly linked with predictable sensory outcomes - a process known as action–perception coupling (Novembre and Keller, 2014; Chang et al., 2025). This integration of motor actions with sensory feedback is a central mechanism in motor sequence learning, supporting error detection, online movement correction, and the reinforcement of accurate spatiotemporal patterns (Diedrichsen and Kornysheva, 2015; Maes et al., 2014; Wolpert et al., 2011; Popp et al., 2022). This supports the gradual refinement of individual movement elements and their integration into a coherent sequence, ultimately resulting in fluent motor sequence performance. Auditory feedback represents a potential method for augmenting motor action – perception coupling (Sigrist et al., 2013; Han et al., 2024; Dyer et al., 2017a; Effenberg et al., 2016). This feedback modality can deliver sensory information related to both timing and spatial (i.e., pitch) aspects and has been shown to influence motor performance across various tasks by modulating timing accuracy and/or spatial precision - particularly when the feedback was informative or motivational (Lappe et al., 2018; Han et al., 2024; Bangert et al., 2006; Luciani et al., 2022; Leow et al., 2025; Stöcker and Hoffmann, 2004; Rosati et al., 2012; Dyer et al., 2017a; Keller and Koch, 2008; Rusconi et al., 2006; Silva et al., 2017). However, external feedback can create dependency on the feedback source, causing learners to disregard their internal feedback. Several studies show that while sensory feedback enhances initial motor learning, it may hinder the development of feedback-independent intrinsic task representations and impair later skill retention when the sensory feedback is removed (Sigrist et al., 2013; Maslovat et al., 2009; Park et al., 2000; Ronsse et al., 1991; Dyer et al., 2017a). Recent research, however, suggests that this so-called guidance effect may especially apply to visual feedback modalities whereas auditory feedback could improve online skill acquisition without impairing retention in the absence of feedback (Danna et al., 2015; Dyer et al., 2017b; van Vugt and Tillmann, 2015; Ronsse et al., 1991; Finney and Palmer, 2003). For example, in professional pianists, auditory feedback enhances immediate performance and also supports long-term retention even when feedback is later removed, suggesting the presence of robust predictive sensorimotor representations that enable accurate performance independent of auditory feedback (Finney and Palmer, 2003). In contrast, non-musicians seem to depend more on congruent auditory feedback, likely due to their limited experience in mapping sounds to motor actions that produce them (Luciani et al., 2022). This raises the question of whether non-musician learners might also benefit from augmented auditory feedback not only during initial online motor skill acquisition, but also in terms of offline motor memory consolidation and subsequent recall in the absence of the auditory feedback provided during training.

This exploratory study investigates how congruent pitch feedback - providing a consistent mapping between tones and movements - compares with two uninformative forms of auditory feedback, a fixed single tone and random pitch feedback, in shaping online and offline motor sequence learning in healthy young adults without musical training. Congruent pitch feedback was considered informative because it offered predictable, spatially meaningful feedback, whereas fixed feedback (a single, predictable tone) and random feedback (unpredictable tones) were regarded as uninformative. We assessed within-session (online) performance gains and between-session (offline) consolidation after a delay to clarify the role of pitch feedback across different learning phases. We hypothesized that predictable informative congruent pitch feedback would enhance online learning relative to uninformative fixed or random pitch feedback by augmenting sensorimotor coupling. Furthermore, if congruent pitch feedback supports the formation of intrinsic, sequence-specific motor memories, its benefits should persist during delayed retesting even in the absence of feedback. Conversely, if its effects are limited to online learning, benefits would be restricted to real-time error correction or motivational support without lasting impact on consolidation. A clearer understanding of how pitch feedback influences motor memory formation may inform its use as a practical tool to support motor learning, for instance in rehabilitation of motor function impairments following brain injury.

## Materials and methods

### Ethical standards

The study protocol was approved by the local ethics committee of the Medical Faculty of the University of Leipzig (registration code: 047/22-ek). Written informed consent was obtained from all participants prior to the commencement of any study-related procedures.

### Participants

Sixty young, healthy participants aged between 18 and 40 years (mean age 28.4 ± 4.6 years; 25 female) were recruited through local advertisements at University of Leipzig Medical Center. No participant had received more than one year of formal musical training or was currently learning an instrument. None reported a history of neurological or psychiatric disorders, including alcohol or other substance abuse. All participants were screened for cognitive impairment using the Montreal Cognitive Assessment (Nasreddine et al., 2005; exclusion cut-off < 26) and for symptoms of depression using the Beck Depression Inventory (Beck et al., 1961, exclusion cut-off > 19). The Stanford Sleepiness Scale (Hoddes et al., 1973) was administered prior to the morning training session and the retest sessions in the afternoon to assess subjective vigilance at the time of task performance. All participants performed the task with their right hand. The sample comprised 53 right-handed (88.3%) and seven left-handed (11.7%) individuals.

### Task and experimental procedure

**FIGURE 1:**
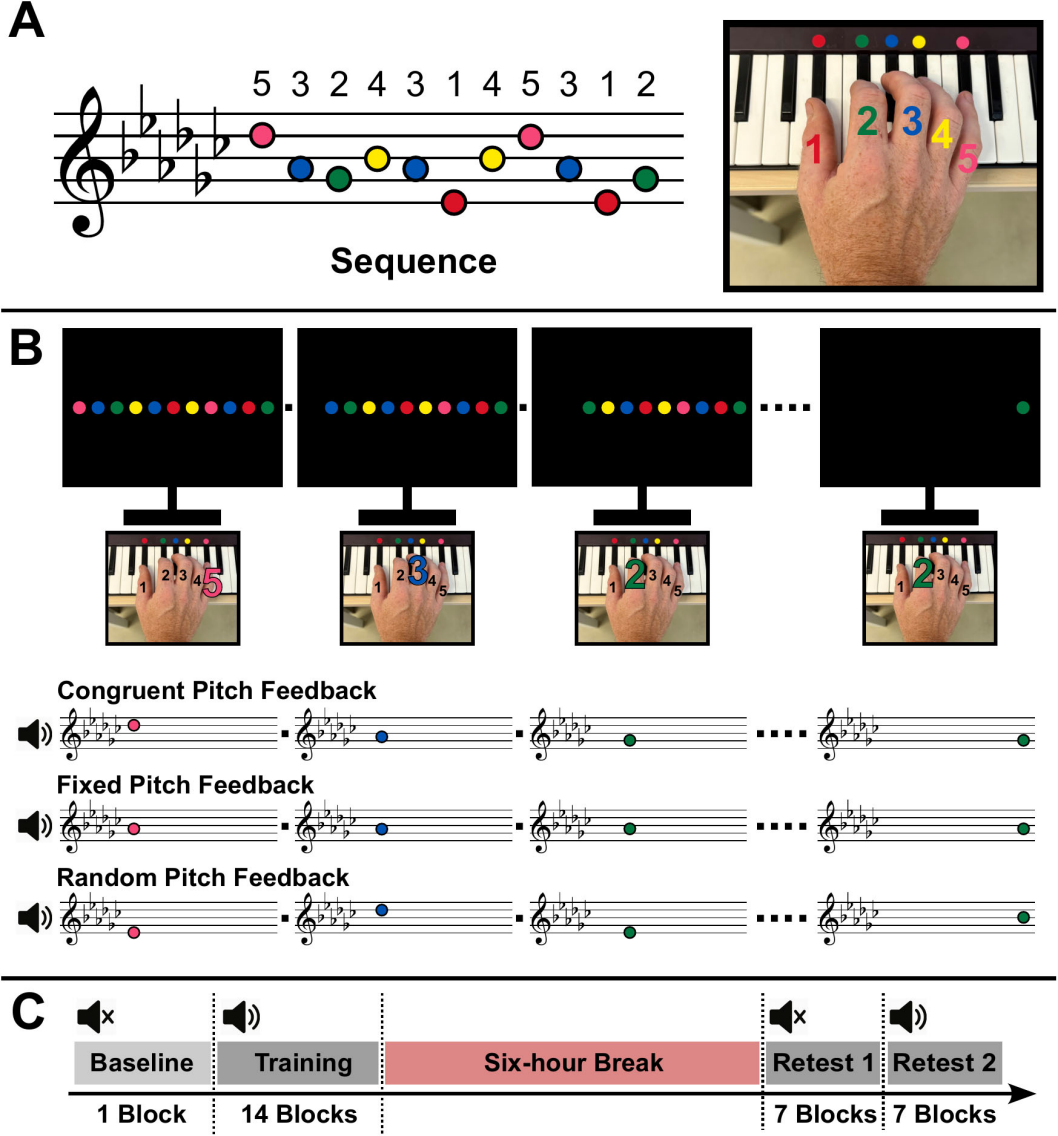
Experimental design, task, and procedure. **(A)** Left side: sequence of notes from the G♭ major/E♭ minor pentatonic scale with assigned fingering (5 = little finger, 4 = ring finger, 3 = middle finger, 2 = index finger, 1 = thumb), right side: placement of the right hand of participants on the keyboard, keys used for sequence execution are marked by distinctly colored dots. **(B)** Illustration of the experimental setup. During the baseline block and each block of training or retesting, the sequence was visually presented on a computer screen as a horizontal array of eleven colored dots, each corresponding to the color-matching key on the keyboard. Participants were instructed to press the keys in the order (from left to right) as indicated by the visual cues. When a correct key was pressed, the corresponding dot disappeared, indicating progression to the next item. An incorrect key press caused the display to reset to the full horizontal array of 11 colored dots, signaling to start over. In the congruent pitch feedback condition, each keystroke produced its corresponding tone from the G♭ major/E♭ minor scale; in the fixed pitch feedback condition, all keystrokes produced the same tone (A♭’); and in the random pitch feedback condition, each keystroke triggered a random tone from the sequence. **(C)** Overview of the experimental design. A baseline block without auditory feedback was followed by the training session (14 blocks) with auditory feedback. Retesting was done after a 6-h break first in the absence of auditory feedback (Retest 1, seven blocks) and then with reinstated auditory feedback (Retest 2, seven blocks).

Participants were randomly assigned to one of three experimental groups, each corresponding to a distinct type of pitch feedback: congruent, fixed, or random. They were informed that the goal of the task was to perform a repeating 11-item sequence of finger-tapping movements on a MIDI keyboard (Reface DX, Yamaha, Shizuoka, Japan) as quickly as possible while making as few errors as possible. All experimental procedures were implemented and controlled using customized software developed in MATLAB 2019b (MathWorks, Natick, MA). MIDI device recordings were handled via the Audio Toolbox, and feedback was presented through a GUI built with the Psychophysics Toolbox extensions (Version 3) (Brainard, 1997; Pelli, 1997; Kleiner et al., 2007). The 11-item finger-tapping sequence consisted of tones drawn from the G♭ major/E♭ minor pentatonic scale (i.e., D♭”–A♭’–G♭’–B♭’–A♭’–E♭’–B♭’–D♭”–A♭’–E♭’–G♭’; Figure 1A), such that only the “black keys” of the MIDI keyboard were used. This tonal sequence corresponded to a fixed finger-tapping pattern: 5–3–2–4–3–1–4–5–3–1–2, where 5 = little finger, 4 = ring finger, 3 = middle finger, 2 = index finger, and 1 = thumb. Each key on the MIDI keyboard involved in the sequence was marked with a distinct colored dot. Participants were instructed to position their right hand on the keyboard such that the thumb (1) was placed on the E♭’ key (red), the index finger (2) on the G♭’ key (green), the middle finger (3) on the A♭’ key (blue), the ring finger (4) on the B♭’ key (yellow), and the little finger (5) on the D♭” key (pink) (Figure 1A).

The sequence was visually presented on a computer screen as a horizontal array of 11 colored dots, each corresponding to their color-matching key on the keyboard. Participants were instructed to press the keys in the order (from left to right) indicated by the visual sequence. When a correct key was pressed, the corresponding dot disappeared, signaling progression to the next item (Figure 1B). If an incorrect key was pressed, the sequence reset, and the entire visual sequence was displayed again to indicate that participants should restart the sequence from the beginning. Participants were able to see their hand while performing the task. Auditory feedback was delivered via the MIDI keyboard’s built-in speakers (sound preset: “LegendEP” with fixed velocity).

To familiarize participants with the task, they were first asked to very slowly perform the sequence without auditory feedback. To confirm successful mapping of visual cues to keypresses, participants were required to perform three consecutive correct sequences before proceeding to the actual experiment. The main experimental session began with a baseline block, during which participants were instructed to perform the eleven-item sequence six times as quickly and accurately as possible, without pitch feedback. This was followed by 14 training blocks. During these blocks, each keystroke triggered one of three types of pitch feedback, depending on the assigned group. “Congruent pitch feedback” group: each key produced its corresponding pitch from the pentatonic scale, providing predictable and spatially meaningful mapping between tones and key positions feedback; “Fixed pitch feedback” group: each keystroke produced the same fixed pitch (A♭’) regardless of which key was pressed, providing predictable, but non-informative auditory feedback; “Random pitch feedback” group: each keystroke triggered a randomly selected pitch from the sequence, providing non-informative unpredictable auditory feedback (Figure 1B). Each block of task execution was terminated after 66 keystrokes, allowing participants to complete a maximum of six correct sequences per block. The onset of each training block was marked by the appearance of the horizontal visual cue array on the screen. The end of a block was indicated by the disappearance of the cue array and the appearance of a red cross. A fixed inter-block interval of 10 s was used. Retention was assessed 6 h later in two phases: Retest 1: seven blocks of sequence execution without auditory feedback; Retest 2: seven additional blocks with auditory feedback reinstated (Figure 1C).

### Data acquisition and analysis

Customized MATLAB scripts were employed to record the timing of keystrokes on the MIDI keyboard and to later compute speed and accuracy metrics for sequence performance. Accuracy was operationalized as the number of correct sequences per block. Speed was measured as the average time (in seconds) required to execute a correct sequence within a given block of task execution (Time to perform a Correct Sequence, TCS). To account for interindividual differences in baseline task performance, TCS values from the training and retest blocks were normalized to each participant’s baseline performance (measured in the absence of pitch feedback) to assess training-induced performance gains using the following formula:


$$\mathrm{normalized}{\mathrm{TCS}}_{x}\left(\mathrm{nTCS},\%\mathrm{change}\mathrm{from}\mathrm{baseline}\right)=$$

$$\left({\mathrm{TCS}}_{\mathrm{Baseline}}-{\mathrm{TCS}}_{x}\right)/{\mathrm{TCS}}_{\mathrm{Baseline}}\times 100$$

where x = block of trials.

To examine differences in online learning during the initial training session as a function of pitch feedback type, we conducted a repeated-measures ANOVA (rmANOVA) on normalized TCS (nTCS) with Block (1–14) as the within-subject factor and Group (congruent, fixed, random pitch feedback) as the between-subject factor. Group differences at the predefined End-of-Training phase (EoT, last four blocks of the training session) were further assessed by an rmANOVA restricted to blocks 11–14. This analysis also allowed us to test for asymptotic learning at EoT, which served as the baseline for assessing offline performance changes between sessions (EoT → Retest 1, EoT → Retest 2). Differences in retention across groups in Retest 1 (without pitch feedback) and Retest 2 (with pitch feedback) were examined using separate rmANOVAs with Blocks (1–7) as the within-subject factor and Group (3 levels) as the between-subject factor. All rmANOVAs were tested for violations of sphericity and Greenhouse–Geisser corrections were applied where necessary, with adjusted degrees of freedom and p-values reported. For main effects of Group, pairwise group comparisons were conducted with corrections for multiple testing (three comparisons) using the false discovery rate (FDR) method (Benjamini and Hochberg, 1995).

Between-session offline skill performance changes were calculated as the individual nTCS difference between the first block of each retest and the EoT baseline (ΔnTCS = nTCS of block 1 of the respective retest – mean nTCS across EoT). We focused on the first block of each retest to minimize contamination of offline performance changes through additional online learning during retesting. Across group comparisons of demographic characteristics, baseline TCS, baseline accuracy, and predefined between-session performance change measures (EoT → Retest 1, EoT → Retest 2) were tested for normality (Kolmogorov–Smirnov) and conducted using one-way ANOVA or the Kruskal–Wallis test, as appropriate. For all statistical analyses, the significance threshold was set to α = 0.05. Effect sizes for significant results are reported as eta-squared (η^2^) for one-way ANOVAs, partial eta-squared (η^2^_p_) for rmANOVAs, and Cohen’s d for paired *t*-tests. Data in the main text are presented as mean ± standard deviation. Statistical analyses were performed with SPSS^®^ 29 (IBM, Armonk, NY, United States) and MATLAB (Mathworks, Natick, MA, United States).

## Results

There were no significant differences between groups in demographic characteristics (see Table 1).

**TABLE 1:** Demographic characteristics of participants.

Baseline characteristic	Congruent pitch feedback	Fixed pitch feedback	Random pitch feedback	Group differences
N	20	20	20	–
Sex (f/m)	7/13	8/12	10/10	χ^2^(2) = 0.96, *p* = 0.62
Age (years)	28.1 ± 4.4	28.6 ± 4.4	28.5 ± 5.1	*F*(2,57) = 0.07, *p* = 0.93
BDI	3.6 ± 4.6	1.4 ± 2.4	3.0 ± 3.6	*H*(2) = 4.38, *p* = 0.11
ESS	5.1 ± 2.8	4.7 ± 3.1	5.9 ± 3.0	*F*(2,57) = 0.91, *p* = 0.41
MoCA	29.0 ± 1.1	29.5 ± 0.6	29.1 ± 1.0	*H*(2) = 1.65, *p* = 0.44
SSS_T_	2.8 ± 1.3	2.5 ± 0.9	2.7 ± 0.9	*H*(2) = 0.64, *p* = 0.73
SSS_RT_	3.2 ± 1.0	2.7 ± 1.0	3.3 ± 1.0	*H*(2) = 3.12, *p* = 0.21

Demographic and clinical characteristics of participants including sex distribution, and mean score values with standard deviations for age, the Beck Depression Inventory (BDI), Epworth Sleepiness Scale (ESS), Montreal Cognitive Assessment (MoCA), Stanford Sleepiness Scale (SSS); SSS_T_, Assessment before the initial training session; SSS_RT_, Assessment before the retest session). TCS, average time required to produce a correct sequence across a block of practice; SD, standard deviation; n, number; s, seconds; f, female; m = male.

### No significant effects of pitch feedback on accuracy of task performance

The number of correct sequences during the baseline block across all groups amounted to 5.05 ± 0.67 (mean ± standard deviation, SD; out of a maximum of six correctly performed sequences per block) and there were no significant differences between groups at baseline [congruent, fixed, and random pitch feedback; *H*(2) = 0.66, *p* = 0.720].

**FIGURE 2:**
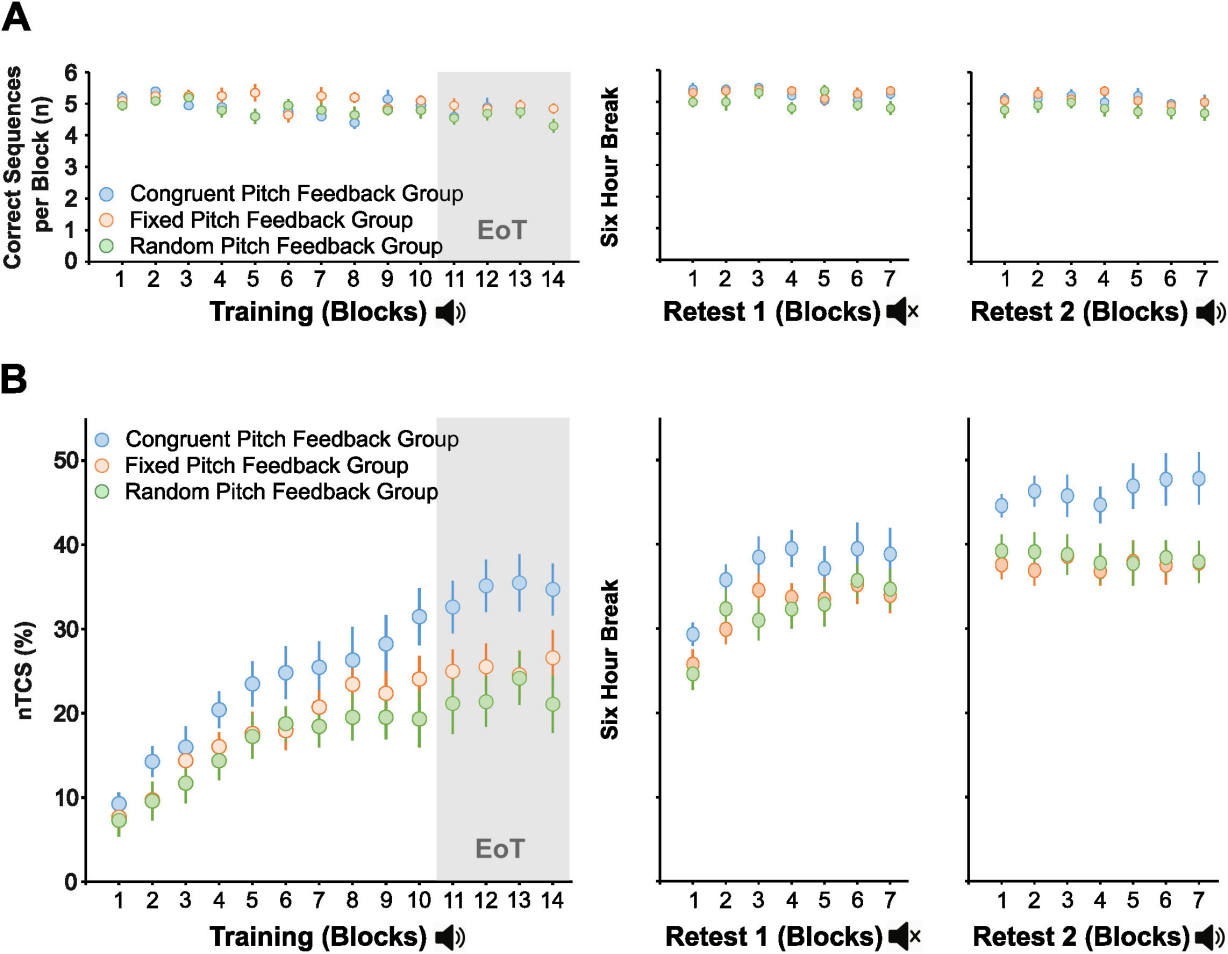
Task performance across initial training and retesting. **(A)** Accuracy: Mean number of correct sequences per block (maximum = 6) across 14 training blocks (with pitch feedback) and delayed retesting after a 6-h break [seven blocks each in Retest 1 (without pitch feedback) and Retest 2 (with pitch feedback)] for the congruent, fixed, and random pitch feedback groups. **(B)** Speed performance: Mean normalized time to complete a correct sequence (nTCS) per block across 14 training blocks (with pitch feedback) and delayed retesting after a 6-h break [seven blocks each in Retest 1 (without pitch feedback) and Retest 2 (with pitch feedback)] for the congruent, fixed, and random pitch feedback groups. Error bars indicate the standard error of the mean. EoT, end of training baseline, nTCS, normalized TCS.

Group differences with respect to accuracy during the initial training session were assessed by a rmANOVA with the between-subject factor Group (congruent, fixed, and random pitch feedback) and the within-subject factor Block across blocks 1–14 of the training session. This analysis revealed a slight but significant decrease in accuracy [rmANOVA, main effect of *Block*: *F*(13,741) = 2.64, *p* < 0.001, η^2^_p_ = 0.044] resulting in a mean number of correctly executed sequences across EoT of 4.76 ± 0.61. This finding likely reflects speed-accuracy trade-off across the training session, given the increasing speed of task performance across blocks of practice (see results below). Importantly, rmANOVA revealed no significant main effect of *Group* [*F*(2,57) = 1.71, *p* = 0.191] nor a significant interaction of *Group* × *Block* [*F*(26,741) = 1.34, *p* = 0.120] across the training session that would indicate a differential modulation of accuracy of motor sequence execution by the type of the pitch feedback intervention. Moreover, rmANOVA computed on the number of correctly performed sequences per block across both retest sessions with the within-subject factors *Session* (Retest 1, Retest 2) and *Block* revealed a slight but significant difference in the mean number of correctly performed sequences per block across Retest 1 (without auditory feedback, 5.19 ± 0.44) vs. across Retest 2 [with auditory feedback, 5.04 ± 0.58, *Session*: *F*(1,57) = 6.82, *p* = 0.012, η^2^_p_ = 0.107]. However, there was again no significant effect of *Group* [*F*(2,57) = 2.66, *p* = 0.078], nor a significant interaction of any factors (all *p* ≥ 0.766) that would point to differences in accuracy between different types of pitch feedback (Figure 2A).

Overall, the findings show that accuracy was high throughout the experiment and consistent across all groups. More importantly, accuracy of task performance was not relevantly modulated by any of the pitch feedback conditions. Therefore, we focused on changes in speed performance (nTCS) as the primary metric to examine the effects of pitch feedback on motor sequence performance.

### Impact of pitch feedback on online learning in the initial training session

In the baseline block without pitch feedback, the average time to execute a correct sequence (TCS) was 7.73 ± 1.84 s (mean ± standard deviation) in the congruent pitch feedback group, 7.23 ± 1.53 s in the fixed pitch feedback group, and 7.14 ± 1.60 s in the random pitch feedback group. Importantly, one-way ANOVA showed no significant differences in baseline performance between groups [*F*(2,57) = 0.74, *p* = 0.483], indicating comparable baseline motor sequence performance across groups. With this prerequisite in place, performance changes during the training and retest sessions were assessed relative to each individual’s baseline performance using normalized TCS values (nTCS; Figure 2B).

Repeated-measures ANOVA (rmANOVA) calculated on the nTCS values across the training session with the within-subject factor *Block* (Block 1–14) and the between-subject factor *Group* (congruent pitch feedback, fixed pitch feedback, random pitch feedback) revealed a significant main effect of *Block* [*F*(4.1, 235.6) = 41.58, *p* < 0.001, η^2^_p_ = 0.422] and *Group* [*F*(2,57) = 3.67, *p* = 0.032, η^2^_p_ = 0.114] in the absence of a significant *Group* × *Block* interaction [*F*(8.3, 235.6) = 1.49, *p* = 0.160]. *Post hoc* pairwise *t*-tests indicated that the significant main effect of Group was driven by higher nTCS across training blocks in the congruent pitch feedback group (25.5 ± 10.9%) compared to the random pitch feedback group [17.4 ± 10.0%, *t*(38) = 0.05, FDR-corrected *p* = 0.033, Cohen’s *d* = 0.78] and the fixed pitch feedback group (19.7 ± 8.4%, FDR-corrected *p* = 0.096). There was no significant nTCS difference across training between the fixed and random pitch feedback groups [*t*(38) = 0.03, FDR-corrected *p* = 0.854].

**FIGURE 3:**
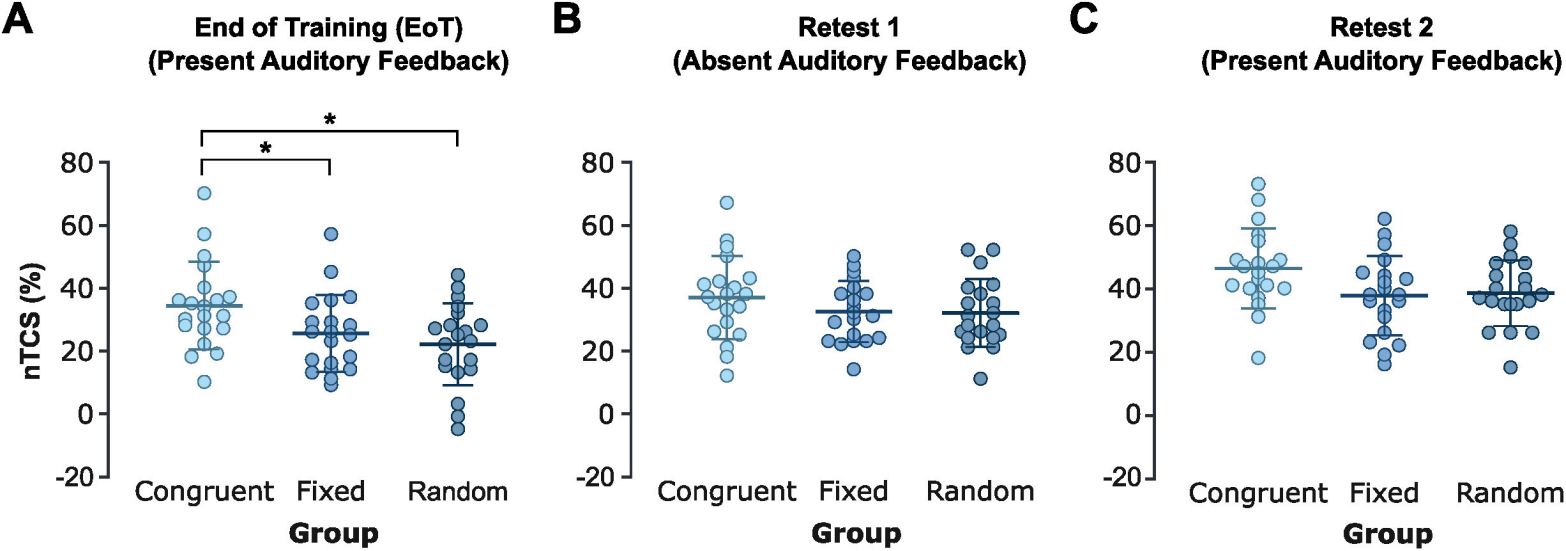
Skill improvements at the end of the initial training session and delayed retest sessions. Mean normalized time to complete a correct sequence (nTCS) for each participant in the three groups (congruent, fixed, and random pitch feedback groups) across. **(A)** End of Training (EoT; last four blocks of the initial training session), **(B)** Retest 1 without auditory feedback (mean nTCS across seven blocks of retesting), and **(C)** Retest 2 with auditory feedback (mean nTCS across seven blocks of retesting). Each dot represents an individual participant; thick horizontal lines indicate the group mean; thin horizontal ticks represent the standard deviations of the group mean. * Indicates FDR-corrected *p* < 0.05. Created in https://BioRender.com.

Across the predefined EoT phase (i.e., last 4 blocks of the training session), rmANOVA confirmed a significant main effect of *Group* [*F*(2,57) = 4.92, *p* = 0.011, η^2^_p_ = 0.147] in the absence of a significant effect of *Block* [*F*(3,171) = 0.97, *p* = 0.409] and absence of a significant interaction of both factors [*F*(6,171) = 0.80, *p* = 0.574], indicating that stable asymptotic learning was reached at the end of the initial training session in all three groups. Pairwise *post hoc t*-tests demonstrated significantly larger nTCS values across EoT in the congruent pitch feedback group (34.5 ± 13.8%) compared to the random pitch feedback group [21.9 ± 13.1%, *t*(38) = 0.02, FDR-corrected *p* = 0.012, Cohen’s *d* = 0.932] and the fixed pitch feedback group [25.4 ± 12.2%, *t*(38) = 0.04, FDR-corrected *p* = 0.048, Cohen’s *d* = 0.693]. The fixed and random pitch feedback groups did not show a significant difference in nTCS across EoT [*t*(38) = 0.13, FDR-corrected *p* = 0.403, Figure 3A].

Overall, this suggests that pairing repeating sequential finger movements with congruent pitch feedback facilitates training-induced performance gains across a single short training session compared to learning the sequence with random or fixed (by trend) pitch feedback.

### Retest 1 – retention in the absence of pitch feedback

A rmANOVA on nTCS across blocks 1–7 of Retest 1 was conducted to assess group differences across delayed retention in the absence of feedback. The analysis revealed a significant main effect of *Block* [*F*(4.2,237.1) = 33.41, *p* < 0.001, η^2^_p_ = 0.370], but no significant main effect of *Group* [*F*(2,57) = 1.19, *p* = 0.312] or *Block* × *Group* interaction [*F*(8.32,237.1) = 1.17, *p* = 0.318], indicating that the motor sequence performance gains facilitated by congruent pitch feedback during training did not generalize to retesting in the absence of pitch feedback (Figure 3B).

### Retest 2 – retention with pitch feedback

When auditory feedback was reintroduced during the second retest, rmANOVA of the nTCS values across blocks of retesting revealed a significant main effect of *Group* [*F*(2,57) = 3.24, *p* = 0.047, η^2^_p_ = 0.102] in the absence of a significant effect of *Block* [*F*(6,342) = 1.07, *p* = 0.383] or a significant interaction of both factors [*F*(12,342) = 1.17, *p* = 0.306]. The significant group effect was driven by an average nTCS across blocks of Retest 2 of 46.3 ± 12.7% in the congruent auditory feedback group vs. 37.6 ± 12.4% and 38.4 ± 10.4% in the fixed and random pitch feedback groups, respectively. However, pairwise *post hoc t*-tests revealed no significant nTCS differences between the congruent and fixed auditory feedback groups (FDR-corrected *p* = 0.063), nor between the congruent and random auditory feedback groups (FDR-corrected *p* = 0.063) after correction for multiple comparisons. There was also no significant difference between the fixed and random pitch feedback groups (FDR-corrected *p* = 0.825, Figure 3C). Overall, this indicates that the superior performance gains associated with congruent auditory feedback during initial training were not maintained (at least not statistically significant) during delayed retesting with reintroduced pitch feedback.

### Impact of pitch feedback on between-session offline performance changes

Offline performance changes across the 6-h break were calculated as the difference between nTCS in the first block of Retest 1 (without pitch feedback) or Retest 2 (with pitch feedback) and the mean nTCS across the EoT phase.

**FIGURE 4:**
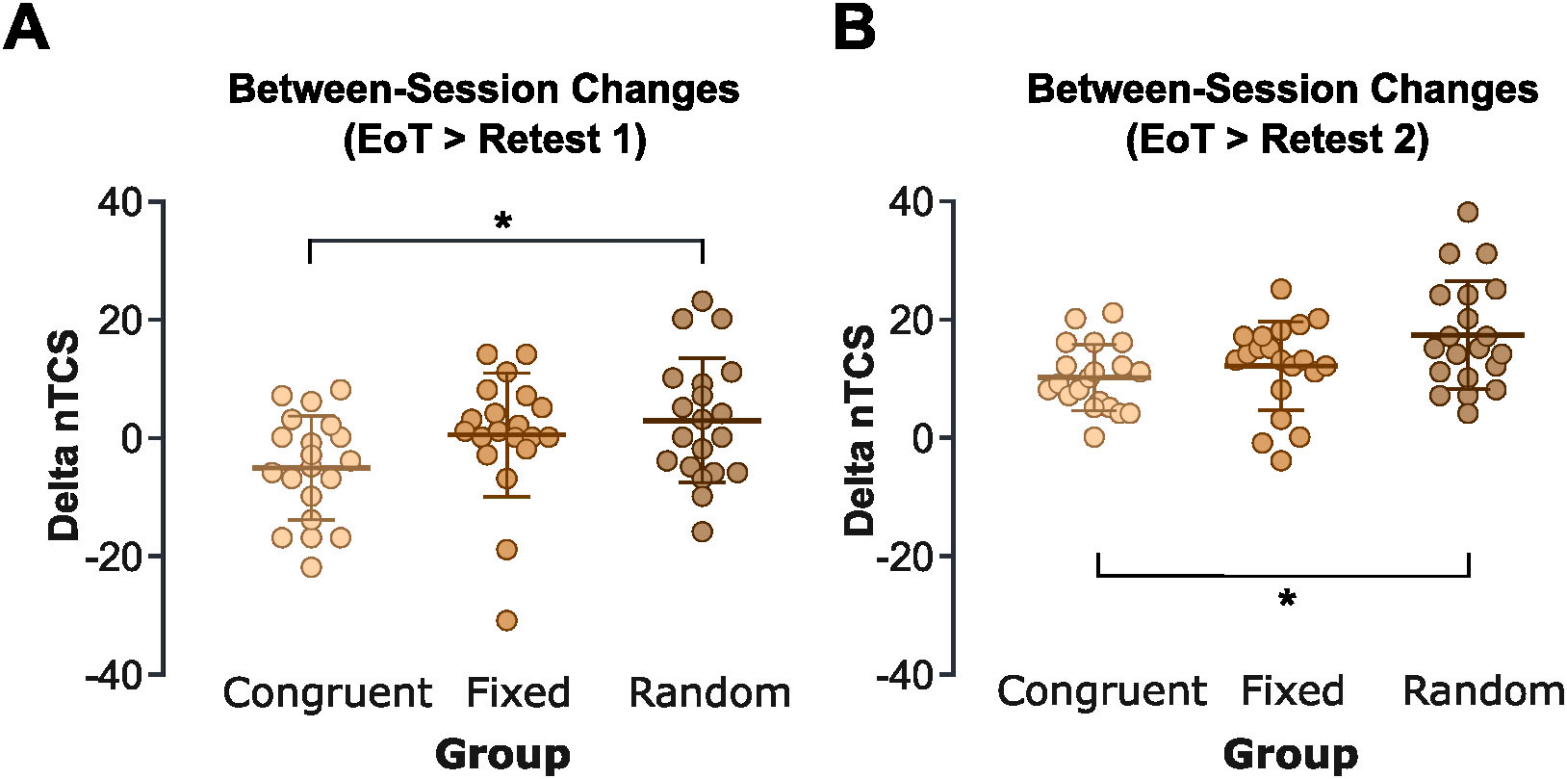
Offline learning. **(A)** Task performance (nTCS) changes between the end of the training session (EoT, mean nTCS across the last four blocks of the training session; with pitch feedback) and the beginning of Retest 1 without pitch feedback (delta nTCS of first block of Retest 1 minus nTCS across EoT) for the congruent, fixed, and random pitch feedback groups. **(B)** Task performance (nTCS) changes between the end of the training session (EoT, mean nTCS across the last four blocks of the training session; with pitch feedback) and the beginning of Retest 2 with reinstated pitch feedback (delta nTCS of first block of Retest 2 minus nTCS across EoT) for the congruent, fixed, and random pitch feedback groups. Each dot represents an individual participant. Positive values indicate between session performance improvements while negative values indicate between-session performance losses. Thick horizontal lines indicate the group mean, thin horizontal ticks represent the standard deviations of the group mean. * Indicates FDR-corrected *p* < 0.05. Created in https://BioRender.com.

A one-way ANOVA on the between-session nTCS differences (EoT → Retest 1) with Group (congruent, fixed, random auditory feedback) as the between-subject factor revealed a significant effect of Group [*F*(2,57) = 3.30, *p* = 0.044, η^2^ = 0.104]. This effect was driven by negative offline performance changes in the congruent pitch feedback group (−5.2 ± 8.8%), compared to slight offline improvements in the fixed [+0.4 ± 10.4%, t(38) = 0.05, FDR-corrected *p* = 0.128] and random [+2.7 ± 10.5%, t(38) = 0.60, FDR-corrected *p* = 0.045, Cohen’s *d* = 0.81] pitch feedback groups. There was no significant difference between the random and fixed pitch feedback groups [*t*(38) = 0.05, FDR-corrected *p* = 0.457; Figure 4A]. This indicates that, while online learning across the initial training session benefited from congruent pitch feedback, these training-induced benefits were lost during retesting in the absence of feedback due to between-session (offline) performance losses. In contrast, participants in the fixed and random pitch feedback groups demonstrated relative offline performance improvements compared to the EoT baseline after withdrawal of pitch feedback.

A one-way ANOVA on the between-session nTCS differences between EoT and the first block of Retest 2 (with reintroduced pitch feedback) revealed a significant group effect [*F*(2,57) = 4.80, *p* = 0.012, η^2^ = 0.143]. Pairwise post-hoc tests indicated that this effect was driven by significantly larger between-session improvements in the random pitch feedback group (17.3 ± 9.1%) compared to the congruent pitch feedback group [10.1 ± 5.6%, *t*(38) = 3.93, FDR-corrected *p* = 0.012, Cohen’s *d* = 0.94]. There was only a trend toward greater gains in the random compared to the fixed pitch feedback group [12.2 ± 7.4%, *t*(38) = 0.57, FDR-corrected *p* = 0.054, Cohen’s *d* = 0.31], and no significant difference between the congruent and fixed pitch feedback groups [*t*(38) = 1.22, FDR-corrected *p* = 0.398; Figure 4B]. Overall, these findings suggest that while congruent pitch feedback enhanced initial online learning relative to random pitch feedback, offline consolidation benefits were significantly greater following training with random pitch feedback.

## Discussion

This study explored the impact of different types of pitch-based auditory feedback on online and offline motor sequence learning in task-naïve non-musicians. Participants who received informative congruent pitch feedback during the acquisition of a novel finger movement sequence showed greater improvements in sequence execution speed during the initial training session compared to those who received uninformative random or fixed pitch feedback. However, pitch feedback had no effect on sequence accuracy. The skill performance advantage in the congruent pitch feedback group persisted numerically but not statistically significant in the retention test conducted 6 h later, when auditory feedback was available. In the absence of pitch feedback, retention was comparable between groups, largely because the congruent feedback group’s performance remained stable compared to the end of training performance, while the groups that received fixed or random feedback showed relative improvements to the performance level at the end of the training session. However, despite demonstrating less improvement in task execution during initial online learning, the group that executed the initial training session with random pitch feedback exhibited significantly greater between-session skill gains compared to the congruent pitch feedback group. This effect was observed at retesting both with and without pitch feedback. These findings suggest a dissociation between feedback conditions that enhance task performance and early online task acquisition versus those that affect offline motor memory consolidation.

Our findings align with existing evidence showing that informative external sensory feedback, such as pitch, can enhance the formation of sensorimotor associations within minutes during the early stages of motor sequence learning in non-musicians (Lappe et al., 2018; Luciani et al., 2022; Pfordresher and Chow, 2019; Bangert and Altenmüller, 2003). In contrast, more experienced learners, such as professional pianists who have already established strong motor–auditory associations, appear to rely less on pitch feedback when learning a novel motor sequence (Luciani et al., 2022; Finney, 1997; Pfordresher, 2012; Pau et al., 2013). Prior research has shown that informative synchronized auditory feedback strengthens sensorimotor coupling and supports consistent movement timing (van Vugt and Tillmann, 2015; Bangert and Altenmüller, 2003), suggesting that the benefits of congruent pitch feedback in non-musicians likely arise from sensory reinforcement of motor patterns through augmenting temporal and spatial structuring. Additionally, these benefits may be linked to the cognitive-perceptual salience of the feedback. When auditory cues are meaningfully mapped onto movement parameters (e.g., an ascending pitch corresponding to spatial position) they enhance error salience and facilitate online monitoring and correction, especially during early learning stages when sensorimotor representations are first established (Sigrist et al., 2013; Effenberg et al., 2016; Bangert and Altenmüller, 2003). Notably, while congruent pitch feedback enhanced initial skill gains compared to uninformative random (significant) and fixed (trend) feedback conditions, no relevant difference was found between the fixed and random feedback groups in the present study. This suggests that congruent pitch feedback facilitates motor sequence learning by strengthening the associations between actions (key presses) and their sensory outcomes (heard pitches), potentially by promoting long-term potentiation (LTP)-like plasticity in motor cortical circuits. In contrast, uninformative (fixed) or inconsistent (random) auditory feedback lacks the structured mapping required to support effective action–perception coupling. Importantly, while previous studies have primarily reported effects of pitch feedback on sequencing error rates (Lappe et al., 2018; Luciani et al., 2022; Finney, 1997; Herrojo Ruiz et al., 2017), our findings suggest no significant effect on accuracy but rather on performance speed. This discrepancy from previous findings may be explained by differences in task demands. In the present study, participants were instructed to execute an 11-item finger-tapping sequence as quickly as possible while being as accurate as possible. In contrast, prior studies often focused on actual musical performance contexts, where participants were required to synchronize their motor actions with a metronome or adhere to a given rhythmic structure (Lappe et al., 2018; Luciani et al., 2022; Finney, 1997). Therefore, our current focus on speed may provide a purer measure of sequence automation that is less contaminated by strategic slowing to avoid errors.

However, although congruent pitch feedback facilitated motor sequence learning and enhanced task performance during initial acquisition, this advantage over random and fixed pitch feedback conditions was no longer evident during the first retention test conducted 6 h later in the absence of auditory feedback. This pattern suggests that while congruent pitch feedback supported immediate performance gains, it was not able to facilitate the encoding and consolidation of feedback-independent intrinsic sequence-specific representations, thereby limiting skill retention when the external feedback is removed. From a theoretical perspective, the formation of internal sequence models is thought to be driven primarily by sensory prediction errors, which enable actions to be evaluated against expected outcomes. With practice, these models may gradually support feedback-independent performance. The loss of the training-induced advantage in sequence performance observed in the congruent pitch feedback group once auditory feedback was removed in Retest 1 indicates that their performance still depended on external auditory cues, suggesting that the underlying sensorimotor representations had not yet consolidated into stable, feedback-independent internal models. In contrast, uninformative inconsistent feedback may have encouraged learners to rely on internal monitoring and sensorimotor prediction, thereby fostering greater independence from external cues during later performance. This interpretation is consistent with the guidance hypothesis (Maslovat et al., 2009; Sigrist et al., 2013; Han et al., 2024; Dyer et al., 2017a), which posits that augmented feedback can enhance immediate performance but may even impair retention if learners become dependent on it. While the guidance effect has been primarily observed and explored in the visual domain, where concurrent visual feedback improves acquisition but reduces retention upon its removal, auditory cues might lead to less feedback dependency due to distinct sensory processing and lower cognitive demands (Eldridge, 2006; Sigrist et al., 2013; van Vugt and Tillmann, 2015; Ronsse et al., 1991; Finney and Palmer, 2003). However, the present findings suggest that non-musicians who have just formed motor action–auditory pitch associations during initial training remain highly dependent on the presence of congruent pitch feedback during retention regarding the surplus gains due to auditory feedback. Notably, although not statistically significant after correction for multiple comparisons, reinstating pitch feedback during the second retention test restored the performance advantage in the congruent pitch feedback group compared to the fixed and random pitch feedback groups, indicating a trend for a lasting benefit in the context of continued presence of pitch feedback.

Our results revealed a dissociation between the effects of pitch feedback on online skill acquisition and offline motor memory consolidation in our task-naïve non-musician participants. Specifically, congruent pitch feedback facilitated early online skill acquisition and improved immediate performance during training compared to uninformative random and fixed feedback, consistent with prior work discussed above showing that meaningful sensory feedback enhances sensorimotor integration (Sigrist et al., 2013; Han et al., 2024; Dyer et al., 2017a; Effenberg et al., 2016; van Vugt and Tillmann, 2015). In contrast, random pitch feedback hindered initial learning significantly compared to informative congruent pitch feedback, likely because its unpredictability disrupted the formation of stable action–perception associations. However, the random feedback group exhibited greater offline skill consolidation between training and both retest sessions (regardless of the presence of pitch feedback during retesting) compared to the congruent feedback group. While the impaired performance observed in the congruent feedback group during the first retest - conducted without auditory feedback - may be accounted for by the guidance hypothesis (i.e., not reflecting impaired consolidation *per se*, but reduced retention due to reliance on the previously available informative feedback), the relatively greater between-session gains observed in the random feedback group during the second retest (conducted with auditory feedback) cannot be attributed to performance impairments in the congruent pitch feedback group due to feedback withdrawal. Instead, this finding suggests that, despite its disruptive effects compared to congruent pitch feedback during initial training, random feedback may have prompted learners to rely more heavily on internal error detection and sensorimotor prediction mechanisms which allow for more efficient encoding and storage of intrinsic sequence specific representations. However, as our study relied exclusively on behavioral measures, this interpretation remains speculative. Future research employing neurophysiological methods (e.g., EEG, fMRI) will be necessary to directly investigate neurophysiological mechanisms underlying these effects.

Several limitations should be acknowledged. First, the study sample consisted solely of healthy, young non-musicians, which limits the generalizability of the findings to other populations. Future research should investigate whether the effects of pitch feedback on motor sequence learning differ in other populations, such as older adults, clinical groups with motor impairments, or expert musicians with extensive prior motor–auditory associations. Second, retention was assessed only 6 h after training, providing limited insight into longer-term consolidation processes that unfold overnight and over days. Third, the study relied exclusively on behavioral measures, leaving the neural mechanisms underlying the observed effects unexplored. Fourth, because retests were always administered in the same order (first without, then with auditory feedback), the magnitude of between-session performance gains at Retest 2 may have been affected by additional practice during Retest 1 and should, therefore, be interpreted with caution. Another limitation of this study is the absence of a true no-auditory-feedback control group. However, our focus was on comparing different types of pitch feedback and completely eliminating any auditory input - including the natural sound produced by striking the keys (e.g., with earplugs) - would have created a fundamentally different task context. Including such a control in future studies could help disentangle the effects of auditory feedback from repetition-based learning alone. It should also be noted that the pitch mappings were predefined and may not have been equally intuitive or salient for all participants, potentially influencing the effectiveness of the feedback across individuals. Moreover, although we do not consider any of the feedback types to be inherently aversive, we cannot exclude the possibility that certain types of feedback - such as unpredictable or repetitive tones - may have been perceived as aversive or monotonous by some participants.

In sum, this study demonstrates that the nature of pitch feedback distinctly modulates motor sequence learning in task-naïve non-musicians, revealing a dissociation between its effects on immediate performance, early online learning and longer-term consolidation. Informative congruent pitch feedback enhanced early online learning compared to uninformative random tone and fixed tone feedback likely by reinforcing motor action–perception mapping and promoting sensorimotor integration. However, this benefit was context dependent and diminished at retesting in the absence of pitch feedback, suggesting a reliance on external cues that may limit the formation of stable, intrinsic motor representations. Conversely, random pitch feedback, despite impairing initial performance and online skill improvements compared to congruent pitch feedback, was associated with enhanced offline motor memory consolidation, potentially by implicitly encouraging learners to engage internal error monitoring and prediction mechanisms. Overall, the limited transfer of feedback-induced performance gains to feedback-free contexts underscores the importance of understanding how different types and schedules of sensory feedback impact motor learning. Our findings suggest that auditory feedback should be tailored to the learning phase: while predictable congruent pitch feedback supports early learning compared to unpredictable auditory feedback, unpredictable feedback may better support long-term retention. This has implications for motor rehabilitation and training programs, where adaptive feedback strategies could optimize both immediate gains and lasting skill acquisition. Future research should explore how to optimize auditory feedback delivery - potentially through adaptive or faded feedback schedules - to promote durable skill learning across sensory contexts.

## Data Availability

The raw data supporting the conclusions of this article will be made available by the authors, without undue reservation. All data underlying the figures are available in the Supplementary Table 1. The scripts used to conduct the experiment and extract the data can be downloaded from https://github.com/ChrisF48/AuditoryFeedbackLearning.git.
